# Evaluating large language models as an educational tool for meningioma patients: patient and clinician perspectives

**DOI:** 10.1186/s13014-025-02671-2

**Published:** 2025-06-14

**Authors:** Diana-Coralia Dehelean, Sebastian H. Maier, Alev Altay-Langguth, Alexander Nitschmann, Michael Schmeling, Daniel F. Fleischmann, Paul Rogowski, Christian Trapp, Stefanie Corradini, Claus Belka, Stephan Schönecker, Sebastian N. Marschner

**Affiliations:** 1 Department of Radiation Oncology, University Hospital, LMU Munich, Marchioninistrasse 15, 81377 Munich, Germany; 2 Bavarian Cancer Research Center (BZKF), Munich, Germany; 3 German Cancer Consortium (DKTK), Partner Site Munich, Munich, Germany; 4 German Cancer Research Center (DKFZ), Heidelberg, Germany

**Keywords:** Meningioma, ChatGPT, Stereotactic radiosurgery, Radiation therapy, Patient experience, Large language model

## Abstract

**Background:**

The study explores the potential of ChatGPT, an advanced large language model (LLM) by OpenAI, in educating patients about meningioma, a common type of brain tumor. While ChatGPT has generated significant debate regarding its utility and ethics, its growing popularity suggests that patients may increasingly use such tools for medical information. The study specifically examines how patients who have undergone radiation therapy for meningioma perceive the information generated by ChatGPT, integrating both patient feedback and clinical assessment.

**Methods:**

Eight meningioma-related questions on diagnosis, treatment options, and radiation therapy were posed to ChatGPT 4. A questionnaire with these responses and feedback items was developed to assess utility, accuracy, clarity, and alignment with patients’ experiences. Nine clinicians first rated each response’s relevance, correctness, and completeness on a five-point Likert scale. Subsequently, 28 patients with meningioma completed the questionnaire during their first follow-up visit (three months post–radiation therapy). Finally, the same questions were presented to three other large language models (ChatGPT 4o mini, Gemini Free, Gemini Advanced), and seven blinded clinicians rated each model’s responses before selecting the most accurate, eloquent, and comprehensive overall.

**Results:**

The study cohort included 28 meningioma patients, mostly female, with a median age of 60 years. Most patients found the information clear, accurate, and reflective of their experiences, with 60% willing to use ChatGPT for future inquiries. Clinicians rated the relevance and correctness of the information highly, although completeness was rated slightly lower, particularly for questions about specific radiation therapy details and side effects. ChatGPT 4 and its newer version ChatGPT 4o mini received the highest, nearly identical scores among the four LLMs evaluated, while Gemini Free scored the lowest in clinician assessments.

**Conclusions:**

ChatGPT demonstrates potential as a supplementary educational tool for meningioma patients, though some areas may require improvement, particularly in providing comprehensive information. The study highlights the potential for integrating AI in patient education, while also noting the need for clinical oversight to ensure accuracy and completeness.

*Trial registration*: LMU ethics vote nr.: 23-0742

## Introduction

ChatGPT is an advanced large language model (LLM) developed by OpenAI© designed to generate human-like text based on the input it receives. (https://openai.com/index/chatgpt/) In the medical field, its potential applications range from streamlining administrative processes to analyzing large datasets faster, integrating in telemedicine platforms or assisting patient education [1]. Many studies have attempted to assess the accuracy of medical information provided by ChatGPT to understand the extent to which it may pose a safety concern [2–4]. Its rising popularity increases the likelihood of patients turning to chatbots for medical inquires, since at least 59% of European citizens use the internet for health-related searches [5].

This study aimed to evaluate how patients who have undergone radiation therapy for meningioma perceived disease-related information generated by ChatGPT. By incorporating both patient feedback and clinical assessment, the study provides a comprehensive evaluation of ChatGPT's potential as a tool for educating meningioma patients in the field of radiation oncology.

Meningiomas are predominantly benign tumors arising from the meninges, the protective layers surrounding the brain and spinal cord. They account for 20% of all primary brain tumors and exhibit a 10-year overall survival of up to 80–90% especially in patients with low-grade meningiomas. Despite the favorable prognosis, the growth and location of meningiomas can significantly impact adjacent brain structures, resulting in a range of symptoms that can impact the quality of life. Treatment primarily aims to relieve acute symptoms or prevent further growth [6].

Given the complexity of treatment options, including surgery or radiation therapy techniques like stereotactic radiosurgery (SRS) and intensity-modulated radiation therapy (IMRT), it is essential for patients to have a clear understanding of their individual treatment plan, its benefits, and potential side effects for ensuring compliance and completing the treatment safely. Radiation therapy typically involves extended treatment courses, with side effects that can emerge even six months post-treatment. For meningioma patients, who often have a long-life expectancy after diagnosis, it is essential to provide proper education to manage the long-term effects of radiation.

## Methods

### Creating the questionnaires to evaluate ChatGPT-generated information on meningioma

Eight disease-related inquiries concerning diagnosis, treatment options and radiation therapy of meningiomas were presented to ChatGPT 4 to generate responses. The final questionnaire included eight responses generated by ChatGPT 4 and five feedback items to score the utility, accuracy and clarity of this information. Additionally, two feedback items were included to score the potential benefits of having access to such information prior to undergoing radiation therapy, and how well the information matched patients' actual experiences (Appendix Table 5, Fig. 3).

### Reviewing quality of information via a questionnaire conducted with clinicians

Prior to patient data collection, each of the eight responses generated by ChatGPT 4 was rated by the nine clinicians from the department of radiation oncology of the LMU Hospital with respect to their relevance, correctness, and completeness on a five-point likert scale. (1-strongly disagree, 2-disagree, 3-neutral, 4-agree, 5-strongly agree).

### Conducting the questionnaire and data collection

28 patients diagnosed with meningioma received the questionnaire during their first follow-up visit after radiation therapy, scheduled three months after the radiotherapy treatment.

Participants were briefed on the study’s objectives and on the confidentiality and voluntary nature of their participation. Written consent was obtained for data collection and analysis. Patients completed the questionnaire in a controlled environment within the healthcare facility. Trained staff was available to assist patients as needed, ensuring a conducive atmosphere for genuine and reflective responses. Data from completed questionnaires were securely compiled for analysis.

### Evaluation of three other large language models

To further explore the quality of medical information provided by large language models, the eight disease-related questions were presented to other LLMs: ChatGPT 4o mini (free version), Gemini Free and Gemini Advanced (Appendix Table 6). Seven experienced clinicians, blinded to the source of the responses, assessed the relevance, accuracy, and completeness of all answers individually using a five-point Likert scale (1-strongly disagree, 2-disagree, 3-neutral, 4-agree, 5-strongly agree). Subsequently, the clinicians reviewed all the responses including the one from ChatGPT 4, to determine which response was the most accurate, eloquent, and comprehensive overall among the four LLMs.

## Results

### Patient characteristics

Patient characteristics are shown in Table 1. The study cohort consisted of 28 participants, the age ranging between 40 and 74 and the median age was 60. The gender distribution was skewed towards females, comprising 68% of the cohort, while males accounted for the remaining 28%. In terms of clinical characteristics 50%, 29% and 4% of the patients were classified as WHO Grade 1, WHO Grade 2 and WHO Grade 3 respectively. In 18% of cases, histological data were unavailable as the diagnosis was based exclusively on radiological findings (MRI). Regarding treatment characteristics, 93% of the cohort underwent normal fractionation therapy, receiving doses of 1.8 Gy (82%) or 2.0Gy (11%) up to a dose of 52.2–60.0, while only 7% received 5.0 Gy per fraction up to 25.0Gy.

**Table 1:** Patient characteristics

Characteristic	Number	Percentage
*Gender*		
Female	8	29
Male	20	68
*Histologic classification*		
No data	5	18
WHO °1	14	50
WHO °2	8	29
WHO °3	1	4
*Fraction dose [Gy]*		
1.8	23	82
2.0	3	11
5.0	2	7
*Total dose [Gy]*		
25 (5 fx)	2	7
50.4 (28 fx)	2	7
52.2 (29 fx)	6	21
54 (30 fx)	9	32
59.4 (33 fx)	6	21
60 (30 fx)	3	11

### Quality assessment with the clinician form

Each question was rated by clinicians regarding its relevance, correctness and completeness. The average Likert scores are listed in Table 2. The majority of the items were rated with an average of ≥ 4 on the Likert scale in terms of relevance and correctness. However, five questions received an average Likert score < 4 in terms of completeness.

**Table 2:** Evaluation of responses generated by ChatGPT 4 to 8 medical questions (Q1–8) related to meningiomas according to experienced radiation oncologists - (Likert scores: 1 - strongly disagree, 2 - disagree, 3 - neutral, 4 - agree, 5 - strongly agree)

	Average Likert score (range)	SD
*Q1: What are meningiomas?*		
Relevance	5 (5–5)	± 0
Correctness	5 (5–5)	± 0
Completeness	4.2 (2–5)	± 0.9
*Q2: What are treatment options for meningiomas?*		
Relevance	5 (5–5)	± 0
Correctness	4.6 (3–5)	± 0.7
completeness	4.3 (4–5)	± 0.5
*Q3: What are the benefits of radiotherapy for meningiomas?*		
Relevance	4.8 (4–5)	± 0.4
Correctness	4.3 (2–5)	± 0.9
Completeness	4.4 (4–5)	± 0.5
*Q4: How is radiotherapy administered to the brain area?*		
Relevance	4.9 (4–5)	± 0.3
Correctness	4.7 (4–5)	± 0.5
Completeness	3.7 (2–5)	± 0.9
*Q5: What is stereotactic radiotherapy?*		
Relevance	4.8 (4–5)	± 0.4
Correctness	4.4 (4–5)	± 0.5
Completeness	3.9 (2–5)	± 0.9
*Q6: What are typical side effects of meningioma radiation?*		
Relevance	4.7 (2–5)	± 0.9
Correctness	4.9 (4–5)	± 0.3
Completeness	3.2 (2–4)	± 0.9
*Q7: What should you pay attention to during radiation therapy to the head?*		
Relevance	4.1 (3–5)	± 0.7
Correctness	4.2 (2–5)	± 0.9
Completeness	3.7 (2–5)	± 0.8
*Q8: What should be considered after radiation treatment?*		
Relevance	4.3 (0.7 3–5)	± 0.7
Correctness	4.4 (1.0 2–5)	± 1.0
Completeness	3.7 (2–5)	± 1.1

### Data collection with the patient questionnaire

**Fig. 1:**
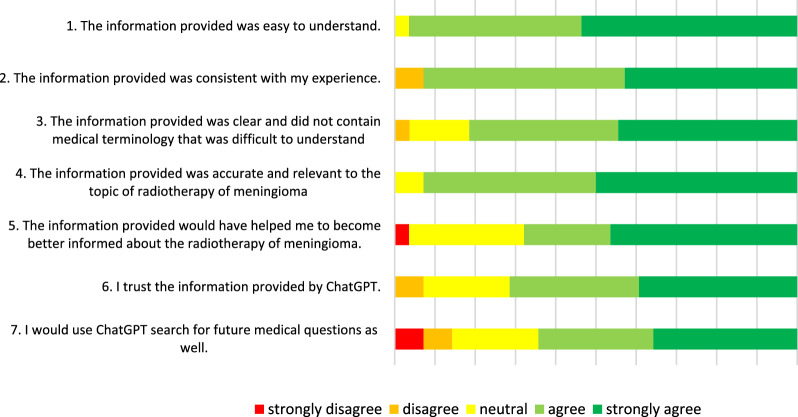
Patient ratings of the responses generated by ChatGPT included in the questionnaire

Over 90% of patients agreed that the information was clear, easy to understand, and accurate. Additionally, over 90% found the information consistent with their own experiences (Fig. 1). Regarding its potential as a prior-treatment educational resource, 65% believed the information would have been helpful in advance, though one patient disagreed, and 31% were neutral. More than 60% of patients trusted the information provided by ChatGPT. Finally, 60% of patients indicated they would use ChatGPT for further medical inquiries, while 19% disagreed (Table 3).

**Table 3:** Patient ratings of the responses generated by ChatGPT: first value indicates patient count, second shows the percentage

	Q1 (N/%)	Q2 (N/%)	Q3 (N/%)	Q4 (N/%)	Q5 (N/%)	Q6 (N/%)	Q7 (N/%)
Strongly disagree	0/0%	0/0%	0/0%	0/0%	1/4%	0/0%	2/7%
Disagree	0/0%	2/7%	1/4%	0/0%	0/0%	2/7%	2/7%
Neutral	1/4%	0/0%	4/15%	2/7%	2/29%	6/21%	6/21%
Agree	12/43%	14/50%	10/37%	12/43%	6/21%	9/32%	8/29%
Strongly agree	15/54%	12 /43%	12/44%	14/50%	13/46%	11/39%	10/36%

### Evaluation of three other LLMs

**Fig. 2:**
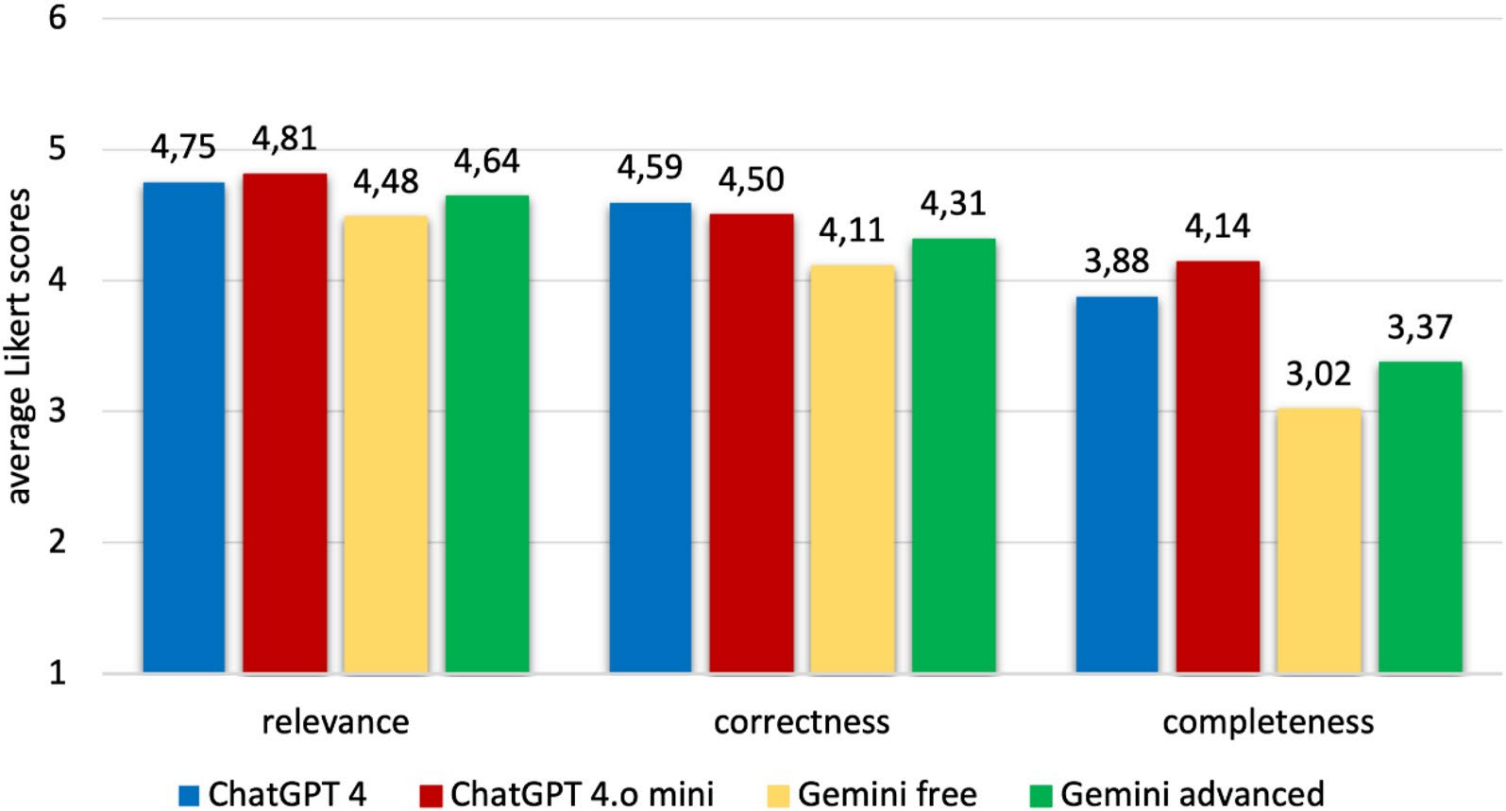
Average Likert scores among the eight disease-related questions presented to different LLMs

**Fig. 3:**
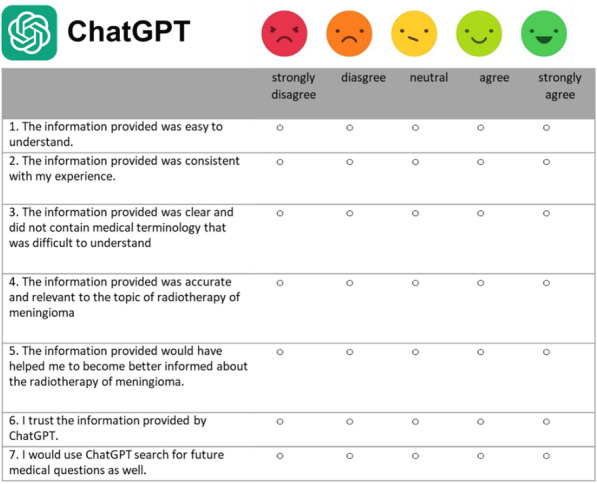
Feedback form presented to patients including Likert scala (translated from German to English)

Among the four LLMs evaluated, ChatGPT 4 and its newer version, 4o mini, received the highest and nearly identical scores, while Gemini Free scored the lowest based on clinician assessments (Figure 2).

At least 50% of clinicians chose one of the two ChatGPT versions from the four options for each question as the best LLM. Gemini was less frequently the first choice with Gemini free being the least popular among clinicians (Table 4).

**Table 4:** Percentage of clinicians who chose the different LLMs providing the most appropriate responses in terms of correctness, eloquence and comprehensiveness for each category

	ChatGPT 4 (% of clinicians)	ChatGPT 4o mini (% of clinicians)	Gemini free (% of clinicians)	Gemini advanced (% of clinicians) (%)
*Q1: What are meningiomas?*				
Correctness	100	0	0	0
Eloquence	57.1	14.3	0	28.6
Comprehensiveness	100	0	0	0
*Q2: What are treatment options for meningiomas?*				
Correctness	42.9	42.9	14.3	0
Eloquence	42.9	28.6	28.6	0
compRehensiveness	57.1	42.9	0	0
*Q3: What are the benefits of radiotherapy for meningiomas?*				
Correctness	71.4	28.6	0	0
Eloquence	57.1	28.6	0	14.3
Comprehensiveness	71.4	28.6	0	0
*Q4: How is radiotherapy administered to the brain area?*				
Correctness	71.4	28.6	0	0
Eloquence	28.6	57.1	0	14.3
Comprehensiveness	57.1	42.9	0	0
*Q5: What is stereotactic radiotherapy?*				
Correctness	42.9	57.1	0	0
Eloquence	42.9	57.1	0	0
Comprehensiveness	57.1	42.9	0	0
*Q6: What are typical side effects of meningioma radiation?*				
Correctness	42.9	42.9	0	14.3
Eloquence	28.6	42.9	14.3	14.3
Comprehensiveness	42.9	42.9	0	14.3
*Q7: What should you pay attention to during radiation therapy to the head?*				
Correctness	28	57.1	14.3	0
Eloquence	28.6	42.9	28.6	0
Comprehensiveness	42.9	42.9	14.3	0
*Q8: What should be considered after radiation treatment?*				
Correctness	42.9	28.6	14.3	14.3
Eloquence	57.1	28.6	14.3	0
Comprehensiveness	57.1	28.6	14.3	0

## Discussion

This study aimed to evaluate the potential accuracy and relevance of ChatGPT in addressing medical questions related to meningioma therapy, as perceived by patients who underwent radiation treatment. Additionally, the quality of information was reviewed by clinicians to determine whether ChatGPT could effectively support patient education and decision-making without posing a safety risk.

The current literature has primarily focused on rating ChatGPT’s responses by experts [7–10]. This study introduces also patient evaluations of ChatGPT-generated information on meningiomas. By enrolling patients who have already undergone radiation treatment, the study offers a unique perspective based on their firsthand experience with treatment outcomes and side effects. Patients were asked to review the information after their first follow-up meeting ensuring that acute toxicities had resolved and that they had sufficient time to reflect on their treatment experience. While one patient noted that the vocabulary of ChatGPT4 was difficult to read, most patients rated the answers clear and easy to understand. This may be biased by their prior exposure to medical terminology before and during the treatment. It should be noted that previous studies already described the tone used by ChatGPT as professional and concise [1]. This raises the question of whether its tone contributes to its trustworthiness, since more than 60% of patients enrolled in this study stated that they trust the information received by ChatGPT 4. On the other hand, the fact that about 90% of patients agreed that the information provided was consistent with their own experience surely also contributed to ChatGPT’s trustworthiness. Although patients completed the questionnaire after consulting with our physicians —which may have influenced them to underestimate the value of the LLM—they still gave high ratings to the information provided by ChatGPT-4.

ChatGPT was already investigated in various situations as a tool for support in cancer patients. Its utility expands from quick and free access to medical information to patient-friendly explanations of medical terms or side-effects [3, 8, 11]. This suggests that ChatGPT can serve as an accessible source of concise, relevant information and help simplify medical vocabulary for patient education. Patients in our study agreed that the information provided by ChatGPT 4 about the radiation treatment of meningiomas would have been helpful beforehand, indicating its potential educational role prior to treatment. Decision aids have been shown to be highly effective tools for both physicians and patients in the medical decision-making process [12]. Our findings indicate that ChatGPT could serve as a valuable resource by offering neutral and unbiased information to support shared decision-making. By helping patients gain a balanced understanding of their treatment options, ChatGPT can empower them to participate more confidently in discussions with their physicians. This approach has the potential to significantly enhance patient education, as increased patient involvement in health decisions has been linked to improved medical outcomes [12].

In their review Lleras de Frutos et. al demonstrated that internet use generally has a positive impact on the psychological well-being of cancer patients. However, they also identified forums and social media platforms as major sources of misinformation, which can contribute to confusion among patients. This issue appears to be particularly pronounced among older adults, who reported experiencing higher levels of anxiety and confusion after seeking medical information online. The confusion is likely attributable to the overwhelming volume of unfiltered information as well as the lack of specificity in online resources [5]. Similarly, our study found that 10% of meningioma patients included in the research disagreed with the consistency of information provided by ChatGPT 4 when compared to their personal experiences. This divergence underscores the limitations of standardized medical information in offering a nuanced and comprehensive understanding of individual conditions. To our surprise, ChatGPT refrained from offering very detailed medical advice. While this can be seen as a lack of specificity, it also avoids misinformation by ensuring that critical decisions—such as determining treatment regimens—remain under the purview of qualified medical specialists within the appropriate clinical context. This observation is encouraging and may suggest an ongoing improvement of ChatGPT, since older studies had noted that ChatGPT might also generate fabricated unreal data [13].

Overall, the five potential risks described by Liu et al., such as generating fake medical content, perpetuating bias, and raising privacy concerns, must still be considered when using LLMs. They discussed in their analysis the challenges of training AI systems for workflows in radiology and proposed guidelines for implementation in clinical practice. The authors highlighted issues such as the lack of generalizability, limited reproducibility, and ethical concerns related to data privacy and the potential for biases embedded in training data [14]. On this note, Leon et. al summarized the challenges posed specifically by the use of ChatGPT in the medical sector and proposed several ways of implementing the use of such a LLM safely, among which data protection and clear ethical guidelines [15]. Our study also proved that as LLMs should be used in optimizing the process of patient education only with rigorous professional – and human - oversight.

The additional clinician evaluation conducted in this study revealed that the responses generated by ChatGPT 4 were medically correct and relevant, consistent with findings from other studies on oncology-specific information [7, 10]. However, our clinicians were hesitant to consider the responses generated by ChatGPT 4 regarding radiation treatment of meningiomas as complete, with only 3 out of 8 questions scoring an average of more than 4 on the Likert Scale. Notably, the response concerning the radiotherapy-associated side effects scored the lowest score (3,2). This may be due to ChatGPT’s tendency to provide general answers that do not adequately address the complexity of a certain treatment administered to the brain. Considering the complex anatomy of the central nervous system, the range of expected side-effects both on short and long term can significantly vary based on the exact location of the meningioma. While the answer provided by ChatGPT 4 did not cover the full range of symptoms, it specified that the side-effects depend on the tumor localization. This raises the question of whether ChatGPT could deliver a more specific answer if provided with more detailed information about the localization and size of the meningioma. So far, Haemmerli et. al assessed ChatGPT’s ability to deliver treatment recommendations similar to those of interdisciplinary tumorboards by presenting 10 glioma cases including data regarding the histology, localization and size. While ChatGPT was able to offer general treatment recommendations for gliomas, it failed to specify the radiation and chemotherapy regimen and to consider the patient’s functional status for decision-making [16].

Following the announcement of ChatGPT 4o mini and the rise of other LLMs, we conducted an evaluation comparing the responses generated by three other large language models (LLMs): ChatGPT 4o mini, Gemini free and Gemini advanced. The assessment revealed that while ChatGPT 4o mini neither significantly outperformed nor underperformed its predecessor, both versions of ChatGPT were rated higher than Google's Gemini models—both the free and paid advanced versions—in terms of correctness, relevance, and completeness. The assessment also revealed that over 50% of clinicians selected the ChatGPT versions as the most appropriate for correctness, eloquence, and comprehensiveness.

One limitation of this study is the small cohort size, influenced by various factors such as limited time availability. Moreover, the sample is disproportionately composed of female participants, likely reflecting the higher prevalence of meningiomas among women. While the limited sample size constrains the generalizability of the findings, the study nonetheless provides a foundational basis for future prospective investigations involving larger, more diverse cohorts. Subsequent studies should also consider collecting data on participants’ educational backgrounds and incorporating open-ended questions to facilitate the inclusion of qualitative insights.

A notable strength of this study lies in its combined patient and clinician perspectives, providing a more comprehensive assessment of ChatGPT’s potential in patient education. By evaluating how well AI-generated information meets patient needs for clarity and usefulness—while simultaneously verifying its medical accuracy, completeness, and relevance through clinical review—we can gain deeper insight into how LLMs can be effectively integrated into healthcare. Future research could assess the robustness of large language models (LLMs) in more complex and uncertain clinical scenarios. Specifically, within the context of radiotherapy, the reliability of LLMs may be evaluated in the assessment of acute toxicities. Additionally, LLMs hold potential for integration into decision-support frameworks aimed at facilitating shared decision-making during physician–patient consultations.

Furthermore, the study’s comparison of ChatGPT with three other large language models underscores differences in performance, reinforcing the value of rigorous, multifaceted evaluations in guiding their safe and optimal implementation. Although the patient sample size may be a limitation, this dual-perspective design remains pivotal in ensuring that both user experience and professional standards are addressed.

## Conclusion

This study highlights ChatGPT’s potential as a tool for educating meningioma patients. Patients found the information clear, accurate, and relatable, while clinicians noted limitations in completeness for detailed treatment-specific information. Comparative analysis of ChatGPT and other LLMs revealed that ChatGPT versions consistently performed slightly better in correctness, relevance, and comprehensiveness. Overall, our results demonstrate that ChatGPT can be a valuable tool for patient education, serving as a preparatory resource for patient-provider discussions within the framework of shared decision-making for example. This approach could have the potential to improve patients' confidence and understanding of their condition, ultimately supporting more informed and collaborative healthcare decisions.

## Data Availability

No datasets were generated or analysed during the current study.

## Appendix

See Table 5, Fig. 3, Table 6.

**Table 5:** Questions and the corresponding responses generated by ChatGPT 4 subjected to evaluation

Question	Response generated by ChatGPT 4
1. What are meningiomas?	Meningiomas are tumors that develop from the meninges, the protective layers of tissue that surround the brain and spinal cord. They represent about one-third of all primary brain tumors. Although the majority of these tumors are benign and slow growing, their size and location of growth can cause them to put pressure on critical brain structures. This pressure can cause a variety of symptoms, including but not limited to headaches, visual disturbances, hearing loss, memory problems, seizures, and motor dysfunction. In rare cases, meningiomas can be malignant or develop into more aggressive forms that grow faster and are more likely to recur.
2. What are treatment options for meningiomas?	Treatment depends on several factors, including the size and location of the tumor, the rate of growth, the patient's age and general health, and the specific symptoms. For smaller meningiomas with no symptoms, expectant management with regular monitoring with imaging may be the best option. For larger, symptomatic, or growing meningiomas, surgical removal is usually the goal. If complete removal is not possible due to the location of the tumor or the risk to the patient would be too great, radiation therapy may be used alone or in combination with surgery. In some cases, drug treatments such as hormone therapy, chemotherapy, or targeted therapies may also be considered.
3. What are the benefits of radiotherapy for meningiomas?	Radiotherapy, particularly modern techniques such as radiosurgery and intensity-modulated radiotherapy (IMRT), can play an important role in treating meningiomas that are difficult to reach surgically or in patients who cannot undergo surgery. Radiotherapy can also serve as an adjuvant treatment after incomplete surgical removal to reduce the risk of tumor recurrence. Advantages of radiotherapy include its non-invasiveness and ability to target the tumor, which protects surrounding healthy tissue and reduces the likelihood of neurological damage.
4. How is radiotherapy administered to the brain area?	The process of radiation therapy begins with careful planning, in which imaging techniques such as computed tomography (CT) and magnetic resonance imaging (MRI) are used to determine the exact dimensions and location of the meningioma. Based on these images, an individual treatment plan is created that specifies exactly how the radiation dose should be distributed. The actual treatment takes place in a special room, where the patient lies on a table and wears a custom-made mask to keep the head still during the radiation application. The radiation is administered in several short sessions in which high-energy rays are delivered specifically to the tumor while the patient lies still.
5. What is stereotactic radiotherapy?	Stereotactic radiotherapy is a specialized form of radiation therapy that delivers highly precise radiation in a single high dose or over multiple fractions. It is called stereotactic radiosurgery (SRS) when it is performed in a single session and fractionated stereotactic radiotherapy (FSRT) when the dose is spread over multiple sessions. This method is particularly useful for meningiomas that are in hard-to-reach locations or where surgery is too risky. Stereotactic radiotherapy may also be an option for patients who experience a recurrence of their tumor.
6. What are typical side effects of meningioma radiation?	The side effects of radiation therapy for meningiomas can vary depending on the location of the tumor and the dose administered. The most common short-term side effects include fatigue, local hair loss on the head in the radiation field and skin irritation. In rare cases, swelling in the brain can occur, which can lead to headaches and other neurological symptoms. Long-term side effects can include cognitive changes, hormone imbalances and, in very rare cases, the development of secondary tumors.
7. What should you pay attention to during radiation therapy to the head?	During radiotherapy, patients should be closely monitored by a multidisciplinary team consisting of oncologists, radiotherapists, neurologists and other specialists as appropriate. It is important that patients report any changes in their symptoms or general condition as these may require adjustments in the treatment plan. Good nutrition, adequate hydration and rest are crucial to support the body during the treatment phase. Patients should also be sure to follow their skin care instructions to minimize skin irritation in the radiation area.
8. What should be considered after radiation treatment?	After radiotherapy is completed, regular follow-up is essential to monitor the condition of the meningioma and respond early to any signs of regrowth. Patients should continue to have regular neurological assessments and seek support for cognitive or psychological challenges as appropriate. It is also important to maintain a healthy lifestyle with a balanced diet and appropriate physical activity to promote overall well-being and aid recovery.

**Table 6:** Responses generated by different LLM versions to the eight meningioma-related inquires. The responses were adapted to comply with a full text and translated to English with Google Translate for the purpose of this publication

1. What are meningiomas?	
ChatGPT 4.o	Meningiomas are tumors that arise from the meninges, which are protective layers of tissue that surround the brain and spinal cord. Meningiomas are among the most common primary tumors of the central nervous system, accounting for about 20-30% of all brain tumors. They tend to grow slowly and are mostly noncancerous (benign), although there may be rare, more aggressive forms. Because they grow slowly, they often go undetected for a long time and only gradually cause symptoms. These symptoms depend on where in the brain or spinal cord they grow and how large they become.
Gemini free	A meningioma is usually a benign tumor that originates from the meninges. These coverings protect our brain. Meningiomas usually grow slowly and can reach different sizes. They often only cause symptoms when they are larger.
Gemini advanced	Meningiomas are usually benign tumors that arise from the meninges, the protective layers that surround the brain and spinal cord. Although they usually grow slowly, depending on their size and location, they can put pressure on the brain or nerves, causing various symptoms.
2. What are treatment options for meningiomas?	
ChatGPT 4.o	Treatment for meningiomas depends on the size, location, growth and patient symptoms. For small, slow-growing meningiomas with no symptoms, a "watch-and-wait" strategy is often used. This involves regular MRI monitoring of the tumor to monitor its growth. Surgery is often the first choice, especially if the tumor is causing symptoms or growing. The goal is to remove the tumor completely, which is often possible with meningiomas because of their mostly benign nature. Radiotherapy is often an option if the tumor is inoperable, could not be completely removed or has grown back. It can control tumor growth and relieve symptoms. Stereotactic radiosurgery is a precise, high-dose form of radiotherapy used for small tumors.
Gemini free	The treatment of a meningioma depends on various factors, such as the size, the location of the tumor and the general health of the patient. Many meningiomas can be surgically removed. Radiation is often used if complete surgical removal is not possible or if there is a fear that the tumor will recur. For small, slow-growing tumors without symptoms, regular monitoring may be sufficient.
Gemini advanced	Treatment for a meningioma depends on several factors, such as the size, the location of the tumor, the age of the patient and the presence of symptoms. For small, asymptomatic meningiomas, regular monitoring with MRI scans may be sufficient. Surgical removal is often the first choice, especially for large or symptomatic tumors. Radiotherapy is used when surgery is not possible or complete, or as an additional treatment after surgery.
3. How is radiotherapy administered to the brain area?	
ChatGPT 4.o	Radiotherapy offers several advantages for meningiomas. Unlike surgery, it does not require any incisions or tissue removal. Modern techniques such as stereotactic radiotherapy allow the radiation to be targeted at the tumor, so that surrounding healthy tissue is largely spared. Radiotherapy can stop or slow the growth of tumors that cannot be completely removed surgically or are inoperable. Radiotherapy is a good treatment option for patients who cannot undergo surgery due to age, general condition or location of the tumor.
Gemini free	Radiotherapy can destroy tumor tissue and thus stop its growth or even reduce its size. Compared to surgery, radiotherapy is often less stressful for the patient. Modern radiation treatments allow for very precise radiation of the tumor, thereby sparing healthy tissue. Radiation can be repeated if necessary.
Gemini advanced	Radiotherapy is a gentle treatment without surgical intervention. Modern techniques enable highly precise radiation that protects healthy tissue. Radiotherapy can stop tumor growth or shrink the tumor and thus alleviate or prevent symptoms. Treatment is usually carried out on an outpatient basis without the need for a hospital stay.
4. How is radiotherapy administered to the brain area?	
ChatGPT 4.o	Before radiation therapy, a planning phase is carried out to precisely locate the tumor and determine the dose. This is done using imaging techniques such as MRI or CT. The radiation therapy itself is usually carried out in several sessions over several weeks. Each session lasts only a few minutes, but precise preparation can take some time. The patient lies on a treatment table during the treatment and the head is stabilized using a mask to ensure precise alignment of the beams. The radiation source (usually a linear accelerator) moves around the patient's head and delivers radiation to the tumor in a targeted manner. This radiation damages the DNA of the tumor cells, which stops their growth or causes them to die.
Gemini free	During radiation therapy, high-energy radiation is directed at the tumor. During the treatment, the patient is fixed in the head area with a special mask to ensure precise positioning. The treatment usually only takes a few minutes and is painless.
Gemini advanced	Before treatment, detailed planning is carried out using CT or MRI images to determine the exact position of the tumor and the surrounding structures. The radiation is administered in several sessions over a certain period of time. During the radiation, you lie on a couch and a special machine directs the rays precisely at the tumor. Regular check-ups during and after treatment are used to check the success of the therapy and to identify possible side effects.
5. What is stereotactic radiotherapy?	
ChatGPT 4.o	Stereotactic radiation therapy, also called stereotactic radiosurgery, is a special form of radiation therapy in which very precise, high-dose beams are focused on the tumor. This method is often used for small, well-defined tumors such as meningiomas. It allows for targeted destruction of the tumor without severely affecting the surrounding healthy tissue. A major advantage of stereotactic radiation therapy is that it can be performed in a few sessions (or even just a single session). It requires precise planning and imaging to ensure that the radiation is precisely directed at the tumor.
Gemini free	Stereotactic radiation therapy is a particularly precise form of radiation therapy. The tumor is displayed in three dimensions using imaging techniques (CT, MRI) and the rays are precisely aimed at the tumor tissue.
Gemini advanced	Stereotactic radiation therapy is a highly precise form of radiation therapy in which several beams are focused on the tumor from different directions. This enables a high dose to be delivered to the tumor while sparing the surrounding healthy tissue.
6. What are typical side effects of meningioma radiation?	
ChatGPT 4.o	Side effects may occur but are usually mild and easily manageable. The frequency and severity depend on the radiation dose, the duration of treatment, and the individual patient's tolerance. Many patients feel fatigued during and after treatment. The skin in the irradiated area may become red or tender. Hair loss is common in the irradiated area but is usually temporary. Some patients develop headaches, but these are usually easily manageable with pain medication. In some cases, brain tissue near the irradiated area may swell, which can cause symptoms such as nausea or neurological deficits. This is often treated with medications such as corticosteroids.
Gemini free	Hair loss may occur in the irradiated area, but this usually recovers after treatment is completed. Many patients feel tired during and after treatment. The skin in the irradiated area may be red and irritated. Headaches are a common side effect. These side effects are usually temporary and subside after treatment is completed.
Gemini advanced	Side effects may occur during or after treatment and are usually temporary. Fatigue is a common side effect and may last for several weeks. The skin in the irradiated area may be red, dry or sensitive. Temporary hair loss may occur in the irradiated area. Headaches may occur during treatment and should be discussed with the doctor. In rare cases, neurological symptoms such as numbness or weakness may occur.
7. What should you pay attention to during radiation therapy to the head?	
ChatGPT 4.o	During radiation treatment, it is important to remain calm and follow the treatment team's instructions carefully. A special mask or holding device is used to ensure that the head remains in the correct position during treatment. Patients should immediately report any discomfort or side effects to the treatment team so that they can be treated in a timely manner. The skin in the irradiated area should be treated gently. Avoid aggressive skin care products and protect the skin from direct sunlight.
Gemini free	During treatment, regular check-ups are carried out to monitor the progress of therapy and to detect possible side effects at an early stage. Skin in the irradiated area should be carefully cared for. It is important to get enough rest and eat a healthy diet.
Gemini advanced	It is important to follow all of the doctor's instructions regarding preparation and treatment. During treatment, get enough rest and avoid physical exertion. Use mild skin care products and protect the irradiated skin from sunlight. Talk to your doctor about any complaints or questions you may have.
8. What should be considered after radiation treatment?	
ChatGPT 4.o	Tiredness may persist for some time after treatment. It is important to take it easy and get enough sleep. The irradiated area may be sensitive even after treatment has finished, so skin care should continue to be gentle and cautious. Regular check-ups are important to monitor the success of the treatment and to detect possible long-term effects in good time. A balanced diet and sufficient fluids contribute to a faster recovery. If symptoms such as severe headaches, nausea or neurological disorders occur after radiation, medical advice should be sought immediately.
Gemini free	After treatment is completed, regular follow-up examinations are important to monitor the treatment results and detect possible long-term effects at an early stage. A healthy lifestyle with a balanced diet and sufficient exercise can support well-being.
Gemini advanced	Keep your appointments for check-ups to check the success of the treatment and to detect possible long-term effects early. Make sure you eat a healthy diet, get enough exercise and sleep to support your recovery. If necessary, you can seek psychological support to deal with possible emotional stress.

## Abbreviations

AI: Artificial intelligence
GPT: Generative pre-trained transformer
IMRT: Intensity-modulated radiation therapy
LLM: Large language model
SRS: Stereotactic radiosurgery
